# Using an Automated Operant Conditioning Procedure to Test Colour Discrimination in Two Juvenile Piranhas, *Pygocentrus nattereri*: A Lesson on Failures and Pitfalls and How to Avoid Them

**DOI:** 10.3390/ani14223187

**Published:** 2024-11-06

**Authors:** Christian Agrillo, Alessandra Pecunioso

**Affiliations:** 1 Department of General Psychology, University of Padova, Via Venezia 8, 35131 Padova, Italy; alessandra.pecunioso@phd.unipd.it; 2 Padua Neuroscience Center, 35131 Padova, Italy

**Keywords:** response learning, place learning, Skinnerian conditioning, red-bellied piranha, colour discrimination, extensive training, cognitive abilities

## Abstract

Most studies on the cognitive abilities of fish have focused on model organisms adopted in behavioural neuroscience. To date, little attention has been devoted to characiformes fish and we record a lack of cognitive investigation on the piranha. In this study, we conducted a preliminary set of experiments to assess whether red-bellied piranhas (*Pygocentrus nattereri*) can solve an automated operant conditioning task, specifically, a reversal learning task. In Experiment 1, the fish were required to discriminate between red and green, while in Experiment 2, they had to discriminate between white and yellow. In either case, we found no evidence of learning capacities with our protocol after extensive training exceeding one thousand trials overall. In Experiment 3, we simplified the learning task by using achromatic stimuli (black and white discrimination) and always presenting the reinforced stimulus on the same side of the tank (a combination of response learning and place learning). Subjects did learn how to discriminate between the colours, although no subject was able to reach the criterion in the subsequent reversal learning task, suggesting that piranhas may be limited in their cognitive flexibility. However, our training procedure may have been inefficient in addressing this issue. We outline some potential limitations of the current methodology to help to establish a more effective approach for investigating operant conditioning in this species.

## Simple Summary

In recent decades, numerous studies have investigated the cognitive abilities of fish, primarily focusing on species commonly used in neuroscience laboratories. In this study, we examined the learning abilities of a neglected species in the field of cognitive ethology, the red-bellied piranha. We assessed whether these fish could learn a colour discrimination task in an automated operant conditioning chamber. In the first experiment, fish were presented with a red vs. green discrimination task, followed by a yellow vs. white discrimination task in the second experiment. In both cases, the fish did not learn the task. We then simplified the task by presenting achromatic stimuli differing in brightness, always on the same side of the tank. This time, the fish successfully learned how to discriminate between the colours. Our findings suggest that red-bellied piranhas may have limitations in their learning abilities. This study also highlights some limitations of the current procedure. We propose that the automated operant conditioning setup needs adjustments to more effectively investigate the learning capabilities of this species.

## 1. Introduction

In the last century, comparative psychologists have primarily investigated the perceptual and cognitive abilities of mammals (mainly primates) and birds (e.g., pigeons). More recently, attention has been devoted to fish species, finding surprising similarities with mammals and birds concerning several perceptual and cognitive abilities [1,2,3]. Fish were found to show cognitive flexibility [4,5], to use numerical information [6,7,8,9,10], to orient in a complex environment [11,12], and, also, some fish showed evidence of self-recognition [13]. However, even though fish represent approximately 50% of living vertebrates, most of the studies focused on a few species that, for scientific reasons, are commonly available in laboratories, such as zebrafish *Danio rerio* [11,14,15], guppies *Poecilia reticulata* [8,16,17], angelfish *Pterophyllum scalare* [6,18,19], goldfish *Carassius auratus* [20,21,22], and cleaner fish *Labroides dimidiatus* [23,24]. Most studies have focused on model organisms from the orders cypriniformes and cichliformes (e.g., zebrafish, goldfish, and angelfish) while less attention has been devoted to the order of characiformes. This group comprises a wide range of fish species living in different ecological contexts, with the piranha being one of the most well-known examples. However, data about their behaviour are scarce [25,26,27]. Currently, piranhas are not considered a threatened species, although collection, trade, and increased pollution/turbidity of Amazonas pose a risk to their population in the future. Given the ecological diversity and importance of characiformes, it is crucial to address this gap in the research to better understand their behaviour and ecological roles. Filling this gap will enhance our knowledge of these species and contribute to broader studies on fish behaviour.

To shed light on the cognitive abilities of piranhas, we used an automated operant conditioning task. Skinner boxes are commonly used chambers for studying operant and classical conditioning [28]. They are often adopted to investigate the perceptual and cognitive abilities of mammals [29,30], birds [31,32], and recently also fish [33,34]. Unlike most studies on animal behaviour, research using automated chambers often tests a restricted number of subjects, each undergoing hundreds or thousands of trials on a highly constrained and stereotypic task, such as nose/pokes. This approach allows for robust data collection on the species’ learning and cognitive abilities, as well as insights into individual performance and inter-individual variation. This is especially common for species that are not typically kept in large groups in laboratories due to practical constraints (e.g., body size) or because they are not established animal models in neuroscience (e.g., gorilla *Gorilla gorilla gorilla* [35], bear *Ursus americanus* [36], elephant *Loxodonta africana* [37], Grey parrot *Psittacus erithacus* [38], blind cavefish *Phreatichthys andruzzii* [39], bamboo shark *Chiloscyllium griseum* [40]).

In an effort to conduct a preliminary investigation of the cognitive abilities of characiformes fish, we initially employed a colour discrimination task in an experiment with the red-bellied piranha *Pygocentrus nattereri*. The colour discrimination task is one of the most common tasks used in the operant conditioning literature [4,41,42,43]. As the colour vision of piranhas (or characiformes in general) is largely unknown, we opted for pairs of stimuli that involved different cone/rod receptors in humans: red vs. green (Experiment 1), and white vs. yellow (Experiment 2). Since none of the two subjects showed any evidence of learning in either experiment, we set up a third experiment in which we bypassed the issue of colour vision by presenting black and white stimuli. Furthermore, we consistently presented the reinforced stimulus on the same side of the apparatus. This approach aimed to facilitate the task by combining response learning with place learning [44]. Once the learning criterion was reached, we reversed the contingency of reinforcement between the two stimuli. This aimed to assess the cognitive flexibility of fish in a standard reversal learning task [4,45]. Given the strong similarities reported in the literature regarding learning abilities in operant conditioning tasks among teleost fishes [2,3,46], we hypothesized that piranhas would also be able to learn the initial discrimination and then able to solve the reversal learning task. This would align with the idea of there being similar cognitive abilities among distantly related species, supporting the common ancestor hypothesis for cognitive skills in vertebrates [47,48].

## 2. Methods

### 2.1. Subjects

**Figure 1:**
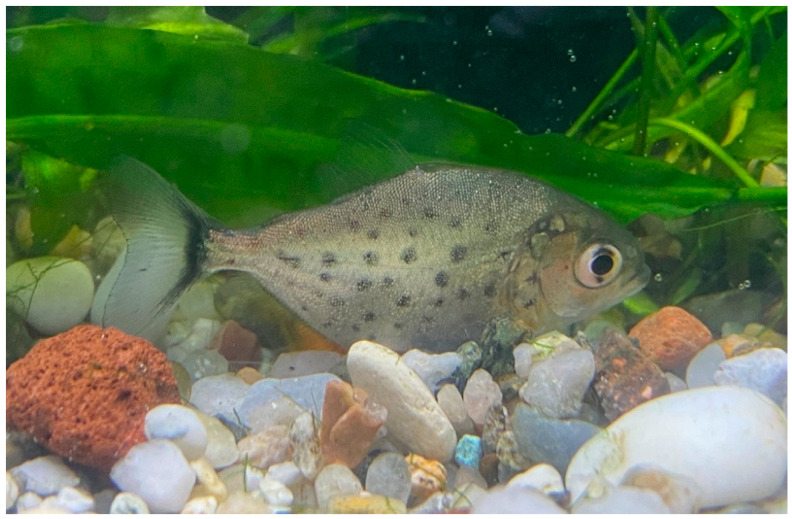
One of the subjects tested in this study: a juvenile red-bellied piranha (*Pygocentrus nattereri*).

Two juvenile individuals (approximately 4–5 months old; total length: 5 cm each) of *Pygocentrus nattereri* were tested (Figure 1). They were sexually immature and were born in captivity in a group of approximately ten individuals. The subjects, named ‘Leo’ and ‘Little José’, were maintained together in a 60 cm × 40 cm × 40 cm (length × width × depth) tank at the Laboratory of Comparative Psychology of the University of Padova in the ten days preceding the beginning of the experiments. Light produced by an 18 W fluorescent bulb was provided above the stock tank (photoperiod was 14:10 h light–dark). The tank also had air filters, natural gravel, and live plants. The water temperature was set to 26 ± 1 °C. Outside of experimental time, fish were fed twice a day, with commercial food flakes in the morning and live brine shrimp (*Artemia salina*) in the afternoon. During day testing, they were fed only in the evening to increase their motivation to obtain food rewards. After each daily session, both subjects were transferred to the stock tank in the Laboratory. Each individual could be easily identified by the experimenters through small differences in the body colours and the morphology of the belly.

### 2.2. Preliminary Assessment of Colour Preference

Before starting the experiment, we needed to assess whether the fish spontaneously preferred some of the colours used in the operant conditioning tasks, which could have biased the learning curves (November 2023).

#### 2.2.1. Stimuli and Apparatus

Each individual was transferred into an experimental chamber in an automated apparatus. The whole unit (Zantiks ©, Cambridge, UK) included the experimental chamber (length × width × depth: 20 cm × 14 cm × 15 cm), a computer, and software using the C++ language. The chamber was made of black plastic walls and a transparent plastic floor and it was filled with 7 cm of water (water temperature: 26 ± 1 °C). A computer screen (20 cm × 14 cm) was placed beneath the tank and used to present two different colours on the floor. A wireless router allowed us to use a laptop to run the programme and collect the data. The system automatically detected the subject’s position through an infrared camera placed above the chamber and an infrared source placed below the chamber. The tank floor was split into two halves: in Test 1, half of the floor was red (RGB: 255, 0, 0) and half of it was green (RGB: 0, 255, 0). In Test 2, the discrimination was between white (RGB: 255, 255, 255) and yellow (RGB: 255, 255, 0). Lastly, we also assessed (Test 3) whether subjects had a preference for white (RGB: 255, 255, 255) or black (RGB: 0, 0, 0) zones (a classical scototaxis test used for assessing the spontaneous preference of fish for bright/dark environments [49,50]).

To further control for side biases, we checked the spontaneous place preferences of the tested fish in the two halves of the tank in a trial where a homogeneous background was presented: a fully red background, a fully green background, a fully yellow background, and a fully white background (Test 4). The procedure was identical to that used in the Tests 1, 2, and 3. Test 4 was run in June 2024.

#### 2.2.2. Procedure

As in previous studies [42,51], after two minutes of acclimation, during which the screen remained blank, we recorded the proportion of time (seconds) spent in the red (Test 1), yellow (Test 2), and black areas (Test 3). The observation lasted 10 min. After 5 min, the position of the two colours was automatically switched by the system to ensure that any potential preference of the fish for one side of the tank was ascribed to the colour/brightness of the background only. For each stimulus pair, we counterbalanced the position of the paired stimuli; for instance, in the first temporal block, Leo had the green area on the left while Little José had the green area on the right.

#### 2.2.3. Data Analysis

Descriptive data (proportion of time spent in one area of the tank) are presented. No inference can be drawn at the population level, given the small sample size and the use of a single trial (a condition necessary to observe the most spontaneous response to the stimuli). These data are provided to offer a qualitative interpretation of the performance of the fish in Experiments 1–3 in terms of a priori preference for our stimuli.

### 2.3. Experiment 1 and 2: Response Learning with Coloured Stimuli

#### 2.3.1. Stimuli and Apparatus

**Figure 2:**
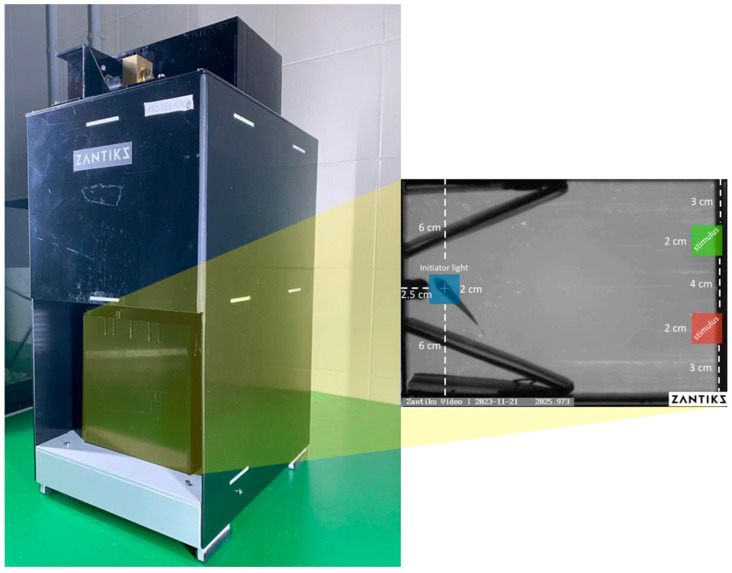
The automated chamber used in this study (Zantiks ©). Subjects were singly inserted into a small tank delimited by black plastic walls. A blue light appeared automatically on the bottom of the tank. To begin each trial, fish had to trigger the disappearance of the blue light. Then, two stimuli (e.g., red and green squares) appeared on the bottom near the other short wall and subjects were required to discriminate between the two colours to receive a food reward.

We used the same automated operant conditioning apparatus described above. We added two plastic walls (0.5 cm × 11 cm × 15 cm) that were aligned with one of the two short walls of the chamber in a funnel-like way to maximize the chance of triggering the disappearance of the initiator light and starting the trial (Figure 2).

Conditioned stimuli consisted of coloured squares (2 cm × 2 cm), the same colours were used in the preliminary assessment of colour preferences: red and green for Experiment 1, yellow and white for Experiment 2. For both experiments, an initiator blue light rectangle (RGB: 0, 0, 255) appeared automatically at the beginning of each trial, on the opposite side of the tank (Figure 2).

#### 2.3.2. Procedure

Each subject was gently inserted into the apparatus with the help of a net. After 2 min of acclimation, a blue initiator light appeared and the trial began. If a piranha swam above that light within 30 s, the blue light disappeared and two coloured rectangles (red and green; yellow and white) appeared on the opposite side of the tank. The inter-stimulus distance was equal to 4 cm. In Experiment 1, Leo was trained to associate the red square with a food reward, while Little José was trained with the green square as the positive (rewarded) stimulus. In Experiment 2, Leo was trained with the yellow square as the positive stimulus and Little José with the white as the positive stimulus.

In the case of the correct choice (i.e., fish swimming above the positive stimulus), the food reward was delivered (2 mg portion of commercial flake food, GVG Sera ©) in correspondence with the blue light that appeared soon after the correct choice (same position of the initiator light). This reward was doubled (approximately 4 mg portion) in Experiment 2 after 100 valid trials, as a preliminary observation of fish performance showed a drastic increase in invalid trials (no choice for either colour) compared to Experiment 1.

If a fish selected the wrong alternative, the two stimuli disappeared, and the bottom of the tank became white until the beginning of the next trial (inter-trial interval: 10 s). If subjects did not select the initiator light within 30 s, or they did not select either stimulus within 30 s, all stimuli disappeared, and the trial was considered invalid. Indeed, each trial could be classified as a valid trial (either correct or incorrect) or an invalid trial (none of the stimuli were chosen or the initiator blue light was not triggered). Each training session consisted of 100 potential trials (valid+ invalid trials overall) except for session 13 for Leo in Experiment 1, which was interrupted after 90 trials due to a technical problem. The position of the stimuli on the left–right axis was switched between trials according to a random sequence. Experiment 1 ended when the fish reached 600 valid trials, to perform more extensive training than the standard operant conditioning procedures used in fish cognitive studies (usually dozens of trials or a hundred trials [1,2,52]). Experiment 2 (March 2024) was run one month after Experiment 1 was concluded (February 2024) and ended when the fish reached 430 valid trials. We needed to interrupt the results of Experiment 2 earlier than Experiment 1, as the number of invalid trials largely increased, thus making it difficult to continue the training. Overall, in Experiment 1 and 2, each piranha underwent 1030 valid trials in the Skinner box.

### 2.4. Experiment 3: Response Learning (Achromatic Stimuli) + Place Learning Task

The subjects, apparatus, and procedure were identical to those used in the previous experiments, but with two important differences: (a) conditioned stimuli were achromatic [black (RGB: 0, 0, 0) vs. white (RGB: 255, 255, 255) discrimination] and (b) stimuli were always presented on the same side of the apparatus. As the background was black too, the borders of the black stimulus were four white lines (0.2 cm). Leo was trained to move towards the black stimulus as the positive (presented on the right side) while Little José was trained to move towards the white stimulus as the positive (presented on the left side). Considering the subjects’ behaviour in Experiment 2, the maximum number of trials for the initial learning (and the subsequent reversal learning task) was fixed at 200 valid trials for the learning discrimination task and 200 valid trials for the reversal learning task. This experiment was run approximately three months after the end of Experiment 2 (mid June/July 2024).

### 2.5. Data Analysis for Experiment 1, 2, and 3.

For all the experiments, the main learning criterion was chosen to be a minimum of 76% of correct choices in the last 50 trials, a criterion previously adopted in training studies in fish [6,53,54] and corresponding to a statistically higher frequency of choices of the rewarded stimulus than the frequency of choices of the unrewarded stimulus in the binomial test (*p* = 0.0003). In addition, as a secondary criterion, we analyzed the frequency of correct choices for each fish in the first 50 and last 50 trials with chi-square tests. If the fish reached one of the two learning criteria in Experiments 1–3, we would have switched the rewarded stimulus and studied reversal learning using the same criteria.

## 3. Results and Discussion

### 3.1. Preliminary Assessment of Colour Preference

**Figure 3:**
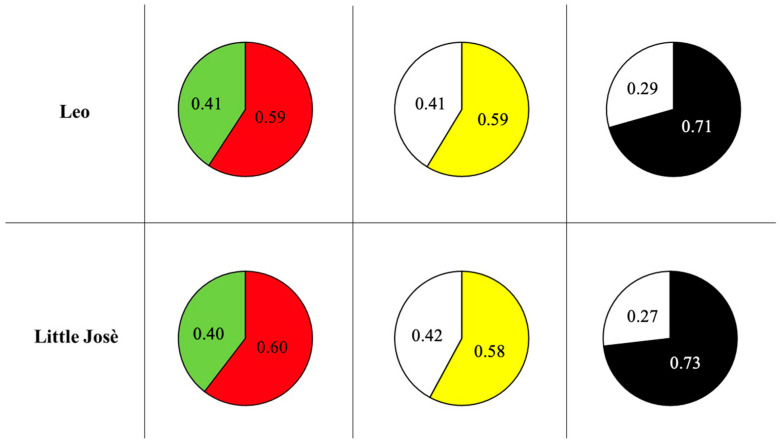
Proportion of time spent by the tested fish in two areas presented to them during the preliminary assessment of colour preference.

Test 1 (red vs. green): Both fish spent approximately 60% of the time in the red area compared to the green one (Figure 3).

Test 2 (yellow vs. white): Leo and Little José spent, respectively, 59% and 58% of the time in the yellow area.

Although these data are only illustrative of possible outcomes (Figure 3), they suggest that fish do not have a preference for any colour, encouraging us to start the operant conditioning tasks without the risk that the spontaneous preferences of the tested fish might facilitate the learning process or interfere with it (e.g., in a red vs. green discrimination task, a preference for red might have helped the subject trained in conditions in which the colour red was positively reinforced).

Lastly, descriptive data obtained in the light/dark preference test (Test 3, scototaxis response) for Leo and Little José suggest a potential preference for the darker area (71% and 73%), which is in line with the robust literature on scototaxis in fish [49,50,55].

**Figure 4:**
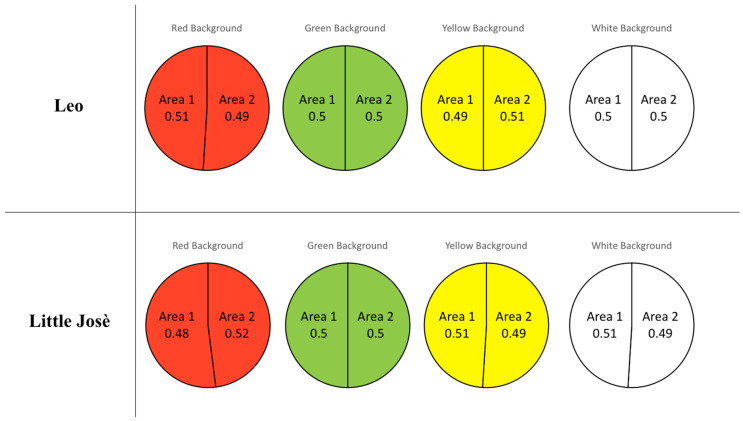
Control test for the preliminary assessment of colour preference (proportion of time spent by the tested fish in the two areas of the same colour).

No evident preference for either side of the apparatus was observed when the same background was presented to the fish (Test 4, Figure 4). This supports the idea that the behaviour of fish observed in the presence of our stimuli is not due to preferences for one side of the tank.

### 3.2. Response Learning with Coloured Stimuli

#### 3.2.1. Experiment 1 (Red Vs. Green)

**Figure 5:**
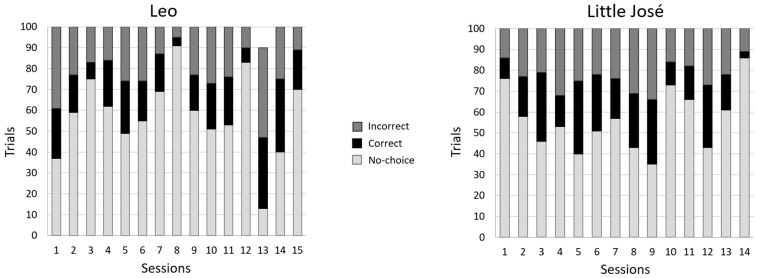
Experiment 1. Numbers of valid (correct/incorrect) and invalid (no-choice) trials for both Leo and Little José.

**Figure 6:**
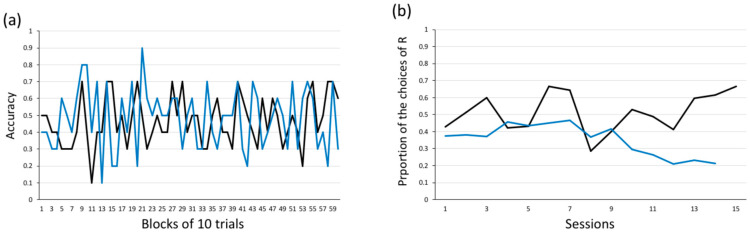
Experiment 1: (**a**) Learning curves. Proportion of correct choices (accuracy) in valid trials, as a function of blocks of 10 trials, for both Leo (black line) and Little José (blue line). Neither of the tested fish reached any of the two learning criteria. (**b**) Side bias: The proportion of choices of the stimuli presented on the right side of the tank as a function of time (successive experimental sessions). While Leo did not show a significant spatial bias, Little José consistently selected the stimulus on the left. R: stimulus on the right side of the tank.

Figure 5 shows the number of valid (correct/incorrect) and invalid trials for each subject. Regarding the learning curves, neither Leo nor Little José reached the learning criterion, nor were they close to reaching it at any stage (Figure 6a). When we compared the frequency of correct choices in the first 50 trials vs. the frequency of correct choices in the last 50 trials (secondary criterion), we did not find a significant difference for either subject (Leo: first 50 trials: 21 correct choices; last 50 trials: 29 correct choices; chi-square test *χ*^2^ = 2.560, *p* = 0.110; Little José: first 50 trials: 20 correct choices; last 50 trials: 19 correct choices; *χ*^2^ = 0.042, *p* = 0.838).

The lack of a significant preference for the reinforced stimulus might have been affected by the side bias that typically emerges in binary tests [51]. To examine this further, we performed a binomial test on the frequency of choices of the stimulus presented on the left and the frequency of choices of the stimulus on the right side. Leo did not show any side bias (binomial test, *p* = 0.172); on the contrary, Little José showed a significant bias by preferentially selecting the stimulus presented on the left side (binomial test, *p* < 0.001). This bias was consistent from the beginning of the training (Figure 6b).

#### 3.2.2. Experiment 2 (Yellow vs. White)

**Figure 7:**
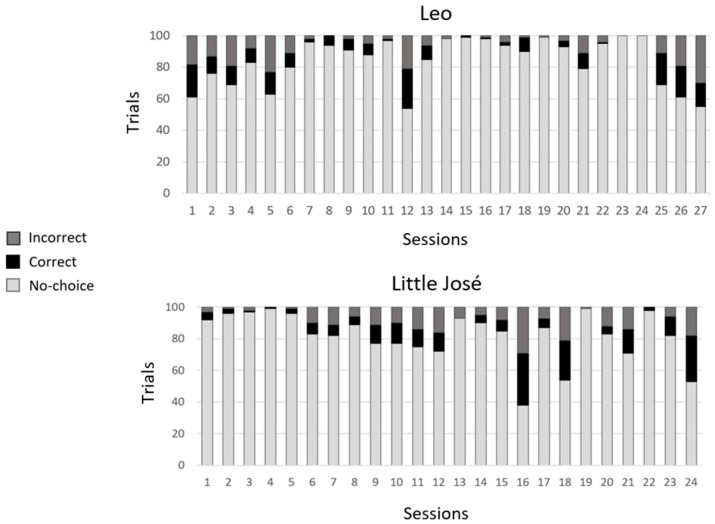
Experiment 2. Numbers of valid (correct/incorrect) and invalid (no-choice) trials performed by both Leo and Little José.

**Figure 8:**
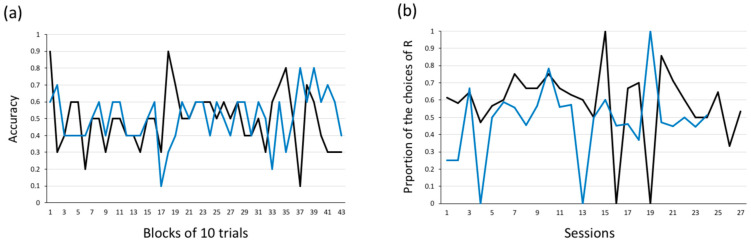
Experiment 2: (**a**) Learning curves. Proportion of correct choices (accuracy) in valid trials, as a function of blocks of 10 trials, for both Leo (black line) and Little José (blue line). Neither of the tested fish reached any of the two learning criteria. (**b**) Side bias. The proportion of choices of the stimuli presented on the right side of the tank as a function of time (successive experimental sessions). While Little José did not show a significant spatial bias, Leo selected the stimulus on the right side of the tank significantly more frequently. R = stimulus on the right side of the tank.

Figure 7 shows the number of valid (correct/incorrect) and invalid trials for each subject. Unlike in Experiment 1, in most of the experimental sessions, both fish performed more invalid than valid trials. Again, neither Leo nor Little José reached the learning criterion, nor were they close to reaching it at any stage (Figure 8a).

When we compared the frequency of correct choices in the first 50 trials vs. the frequency of correct choices in the last 50 trials (secondary criterion), we did not find a significant difference for either subject (Leo: first 50 trials: 28 correct choices; last 50 trials: 19 correct choices; *χ*^2^ = 3.252 and *p* = 0.071; Little José: first 50 trials: 25 correct choices; last 50 trials: 31 correct choices; *χ*^2^ = 1.461 and *p* = 0.227, Figure 8a).

To assess whether there were side biases, we compared the frequency of choices of the stimuli presented on the left vs. right side of the tank by means of binomial tests. This time, Leo showed a right-side bias (binomial test, *p* < 0.001). The bias had been quite consistent since the beginning of the training (Figure 8b). Differently to in Experiment 1, this time Little José did not show any side bias (binomial test, *p* = 0.405).

#### 3.2.3. Experiment 3: Response Learning (Achromatic Stimuli) + Place Learning Task

**Figure 9:**
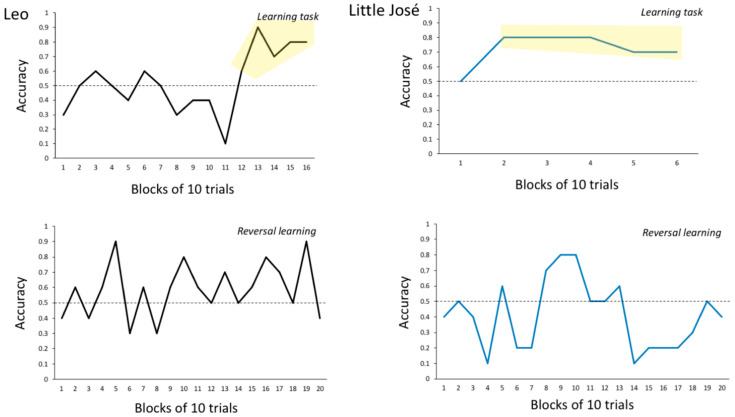
Experiment 3. The learning curves (row above) for the initial discrimination learning (above) and the reversal learning (below) obtained for Leo and Little José. During the initial discrimination learning, the learning criterion was reached in the 50 last trials (the part of the line chart highlighted by the yellow area). Neither fish reached the criteria for the reversal learning.

Leo reached the main learning criterion (76% of correct choices during the last 50 trials) after 160 trials (errors to criterion = 76; invalid trials = 37). When we reversed the contingency of reinforcement between the two stimuli (reversal learning task), Leo failed to reach the criterion after 200 valid trials (Figure 9, invalid trials: 280). Also, the secondary criterion was not reached in the reversal learning task (first 50 trials: 29 correct choices; last 50 trials: 33 correct choices; *χ*^2^ = 0.679 and *p* = 0.410, Figure 9).

Little José reached the main learning criterion after 60 trials (errors to criterion = 20; invalid trials = 52). When we reversed the contingency of reinforcement between the two stimuli, Leo failed to reach the criterion after 200 trials (Figure 9; invalid trials = 102). Also, the secondary criterion was not reached in the reversal learning task (first 50 trials: 20 correct choices; last 50 trials: 16 correct choices; *χ*^2^ = 0.694 and *p* = 0.405; Figure 9).

## 4. Conclusions and Discussion

The first goal of the present study was to conduct a preliminary observation of the learning capacities of red-bellied piranhas (Experiments 1 and 2) using a colour discrimination task. The second goal was to assess their ability to perform a reversal learning task.

Experiment 1 did not show any significant discrimination between red and green, as neither subject reached the learning criteria adopted in this study. In the preliminary assessment, the fish spent approximately 60% of their time in the red area. Although we are aware we cannot make any conclusions in terms of statistical inferences from the preliminary assessment for colour preference, the lack of colour discrimination in Experiment 1 does not appear to be due to a robust bias toward either colour. For instance, if Leo had a preference for red, he might have selected red from the very beginning, regardless of the reward. Similarly, if Little José also had a preference for red, there may have been an initial motivational contrast between selecting the rewarded colour (e.g., green) and selecting the preferred colour (e.g., red). Also, in binary choice tests, it is possible that animals have (or develop across trials) a side bias [51] and thus show an accuracy rate of 50% if stimuli are randomly presented on the left/right side. We assessed whether subjects had a side bias and found that Little José had a left-side bias, which was consistent since the beginning of the training, while Leo did not show any side bias.

In Experiment 2, neither of the two tested piranhas significantly discriminated between white and yellow and did not reach the learning criteria. However, this experiment highlighted a substantial decrease in the subjects’ motivation to make choices during the experimental sessions, with the number of invalid trials exceeding 80%—a drastic increase compared to Experiment 1. We noticed this potential loss of motivation at the beginning of Experiment 2. To motivate subjects to select the stimuli and reach the reward, we doubled the amount of food reward after the first 100 valid trials. Nonetheless, this methodological change did not lead to substantial improvements (or worsening) in performance as the number of invalid trials remained high throughout the experiment. Consequently, we interrupted the training after 430 valid trials, earlier than in Experiment 1. We must point out that there was no sign of learning at any stage, so we believe we did not miss any important information by stopping the experiment before the remaining 170 trials. The decrease in subjects’ motivation may represent a critical issue for extensive training in this species.

It is interesting to note that, in Experiment 2, Leo showed a right bias from the first session, while Little José was bias-free. In contrast, a different pattern of lateralization bias was observed in Experiment 1: Leo was bias free, while Little José was left biased. This indicates that the side biases are not consistent within the automated chamber but emerge independently in each experiment. This aligns with the idea that lateralization biases are context-dependent [56,57,58]. In this case, since the only difference was the colour of the stimuli, we found evidence of a lateralization bias that is stimulus-dependent. Why did this occur? Spiezio and colleagues [58] found that the perception of a red stimulus triggered a motor bias for the use of one hand (the left) in a small monkey, the emperor tamarin, while no hand preference was observed in the presence of a green stimulus. It is possible that some colours (e.g., red and yellow) might trigger a motor bias (e.g., preferential turning to the left or right) in some piranhas too. Alternatively, it has been hypothesized [59] that, in the presence of difficult discriminations, animals might develop a bias (for a given stimulus or a side of the apparatus) in binary choice tests in an attempt to enter the next trial and find the following discrimination potentially easier. In the case of our experiment, regardless of any capacity to discriminate between the two target colours, the consistent selection of a stimulus on the same side of the apparatus led to a food reward in approximately half of the trials. Little José (Experiment 1) and Leo (Experiment 2) might have used this strategy.

Since no evidence of learning was found in the first two experiments, we added a third experiment in which response learning was combined with place learning. Additionally, to avoid any issues related to colour vision, we used black vs. white stimuli. We observed a significant increase in the number of valid trials compared to Experiment 2. As Experiment 3 was run approximately three months after the conclusion of Experiment 2 (an inter-experiment interval three times larger than that of Experiments 1 and 2), we can conclude that the inter-experiment interval is an important methodological variable to consider when carrying out operant conditioning tasks with piranhas. Alternatively, it could be that subjects’ motivation increased in the presence of an easier task, or that the increased age and/or experience of the tested juvenile fish might have been determinant for their performance in Experiment 3. Indeed, this time both piranhas showed the capacity to learn stimuli discrimination, providing the first evidence of learning abilities in an operant conditioning task in this species. In one case, Little José learned very quickly, requiring no more than 60 trials. In the reversal learning task, the fish did not achieve either learning criterion, exceeding the number of trials necessary to learn the initial discrimination. This might reflect a limit in the cognitive flexibility of the species. Alternatively, it could be the result of possible effects of stress on learning and of time-delays in the reversal learning task.

Overall, the results of our three experiments do not allow us to draw any firm conclusions. First of all, studies with small sample sizes may not be sufficiently powered to detect statistical differences, which can lead to false negatives (type II error [60]). Unfortunately, the use of small sample sizes is a common limitation in the field of the cognitive ethology of non-laboratory animals [35,36,37,38,39,40]. We recognize that a larger number of subjects is necessary to provide a broader comprehension of piranhas’ learning abilities, especially considering the significant inter-individual variability in cognitive abilities of various members of the same species [61].

Aside from the problems related to very small sample size, we believe that our study offers better insight into the limitations of the current procedure than into the cognitive abilities of piranhas themselves. It is important to note that subjects were tested in an automated operant conditioning task, which ensures high-throughput conditioning similar to that used with mammals and birds. However, we cannot exclude the possibility that such extensive training may not be optimal for fish. The different metabolic requirements of cold-blooded vertebrates can make food rewards less effective compared to mammals and birds [34]. Fish can be easily satiated and may not need to search for food for extended periods. In our experimental protocol, we ensured that only a small amount of food was provided by the automated chamber. However, after several trials, controlling for olfactory cues in the water becomes difficult, as the smell of food can diffuse throughout the tank. Additionally, while researchers can easily observe whether a monkey consumes its entire reward (usually a pellet in its hand) after a correct trial, in the case of fish, it is challenging to verify whether the subject has completely eaten the food, as it rapidly disperses in the water and is not visible to the naked eye. Consequently, the release of food after numerous trials may have been insufficiently rewarding for the fish.

That said, previous studies using food as reward showed that fish species can solve colour discrimination tasks when placed in a Skinner box (guppies *Poecilia reticulata* [62], zebrafish *Danio rerio* [34]). A recent study compared the performance of guppies when they were trained with a manual task and when they were trained with an automated operant conditioning chamber [63]. The authors found that the automated training device, which models the classical Skinner boxes, can successfully be used to train guppies in certain tasks (i.e., colour, shape, and size discriminations) but is inadequate for others, such as numerical discrimination. This led the authors to suggest that, unlike the traditional manual tasks used in the literature on fish, small laboratory teleosts may be limited in their ability to cope with some undetermined aspects of the automated approach. One important difference between Gatto et al.’s study [63] and our study is that Gatto and colleagues presented their stimuli on a lateral wall of the experimental chamber, while in our case, the stimuli were presented on the bottom of the tank. Although our stimuli are visible and Experiment 3 showed learning in both piranhas with stimuli placed on the bottom, we cannot exclude that this methodological difference might have reduced the fish’s ability to analyze the stimuli. Piranhas are not a bottom-dwelling fish species; therefore, stimuli presented in front of them may be more salient than those presented at the bottom of their environment. Future studies testing our automated chambers with bottom-dwelling fish species are necessary to understand if the current experimental design is more effective with fish accustomed to searching for food on the bottom of rivers or lakes.

Another important difference relates to the area where fish are rewarded. Gatto and colleagues [63] provided food on the side associated with the correct stimulus. In our protocol, when fish selected the correct stimulus, food was released immediately behind them, equidistant from both stimuli where the initiator light was located, to begin the next trial. Although the experimental chamber was small (and the distance between stimuli and reward area was 15 cm), the lack of a strict spatial contingency between the stimuli and reward may have interfered with the learning processes of the piranhas. Other aspects of our protocol might also have been suboptimal. A previous work by Gatto et al. [64] showed that the precision of guppies in training tasks is radically different according to the training setting adopted (e.g., an extension of decision time, whether correction was allowed or not, and 3D vs. 2D stimuli). Future investigation is needed to test whether the 30 s decision time, the absence of corrections for wrong responses, and the use of bidimensional stimuli contributed to the poor results observed in this study.

While our apparatus and procedure require improvements, our results allow us to speculate on piranhas’ perceptual and cognitive abilities. The performance difference between Experiments 1–2 and Experiment 3 may be explained by two factors: (a) piranhas do not distinguish between red and green or white and yellow, or (b) they learn more quickly and efficiently with achromatic stimuli and/or when associating a specific side of the tank with food (place learning). At this time, we cannot definitively support either hypothesis. Although we do not know the spectral sensitivity of this species’ visual system, we consider the ‘perceptual’ hypothesis—lack of colour discrimination in the two discussed experiments—unlikely. Escobar-Camacho and colleagues [65] suggested that characiformes, including the white piranha, possess cones to distinguish red and green. However, no behavioural studies have confirmed the ability of red-bellied piranhas to discriminate between these colours. Regarding the discrimination between yellow and white, it is important to note that yellow involves cones in humans while white also activates rods. Additionally, red-bellied piranhas might distinguish between the two in greyscale, as yellow appears darker than white. Hence, regardless of the distribution of photoreceptors in the piranha’s retina, we might expect some ability to distinguish between these colour pairs in operant conditioning tasks.

The second hypothesis is focused on the relative ease with which piranhas might solve a place learning task. A long-standing debate exists regarding whether animals prioritize one type of learning strategy (for a review see [44]). Research suggests that animals prefer place learning when given a probe trial after limited training, whereas extensive training leads to a predominance of response learning during the probe trial [66,67,68,69]. This suggests that learning the association between a specific location and a reward may be easier than learning to discriminate between two stimuli when spatial cues are absent. If the piranhas’ learning abilities seem limited in our operant conditioning chamber, it may be because they primarily rely on spatial coordinates (e.g., consistently selecting the stimulus presented on the left) rather than learn to respond to specific visual stimuli.

In conclusion, the current automated operant conditioning setup for piranhas requires adjustments to more effectively investigate their learning abilities. Despite these limitations, our study provides preliminary evidence of the learning abilities of red-bellied piranhas through operant conditioning consisting of confronting the tested fish with black and white stimuli which are always presented in the same positions. This highlights the need for further investigations into response learning in this species, which should be distinguished from place learning.

## Data Availability

The data and the script for the automated operant conditioning procedure are available upon request from the corresponding author, C.A.
